# Revealing the interaction mechanism of pulsed laser processing with the application of acoustic emission

**DOI:** 10.1007/s12200-023-00070-7

**Published:** 2023-06-14

**Authors:** Weinan Liu, Youmin Rong, Ranwu Yang, Congyi Wu, Guojun Zhang, Yu Huang

**Affiliations:** 1 State Key Lab of Digital Manufacturing Equipment and Technology, Huazhong University of Science and Technology, Wuhan, 430074 China; 2 School of Mechanical Science and Engineering, Huazhong University of Science and Technology, Wuhan, 430074 China

**Keywords:** Laser dotting, Acoustic emission, Monitoring, Laser ablation, Cracks

## Abstract

The mechanisms of interaction between pulsed laser and materials are complex and indistinct, severely influencing the stability and quality of laser processing. This paper proposes an intelligent method based on the acoustic emission (AE) technique to monitor laser processing and explore the interaction mechanisms. The validation experiment is designed to perform nanosecond laser dotting on float glass. Processing parameters are set differently to generate various outcomes: ablated pits and irregular-shaped cracks. In the signal processing stage, we divide the AE signals into two bands, main and tail bands, according to the laser processing duration, to study the laser ablation and crack behavior, respectively. Characteristic parameters extracted by a method that combines framework and frame energy calculation of AE signals can effectively reveal the mechanisms of pulsed laser processing. The main band features evaluate the degree of laser ablation from the time and intensity scales, and the tail band characteristics demonstrate that the cracks occur after laser dotting. In addition, from the analysis of the parameters of the tail band very large cracks can be efficiently distinguished. The intelligent AE monitoring method was successfully applied in exploring the interaction mechanism of nanosecond laser dotting float glass and can be used in other pulsed laser processing fields.

**Graphical Abstract:**

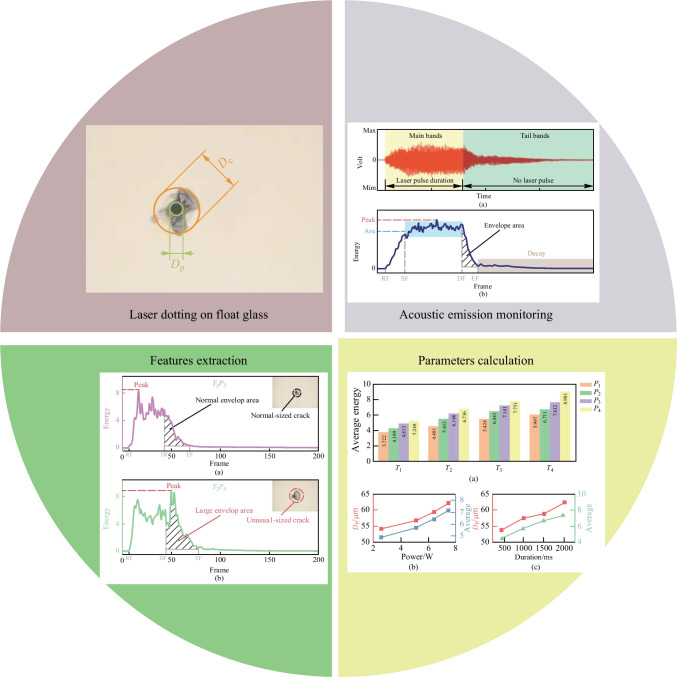

## Introduction

Float glass has excellent physical and chemical properties such as optical transparency, smooth surface, and anti-corrosion [1]. Various laser processing methods have been applied to machining of float glass to meet different needs [2, 3]. For example, laser scribing on float glass is an effective method for forming microfluidics in biomedicine [4], and laser cutting is used to make holes in double glass modules of solar panels [5].

Therefore, the laser processing of float glass has attracted much attention nowadays. Many researchers focus on optimizing parameters to improve the processing quality [6, 7], and others try to build simulation models to reveal the interaction mechanism between laser and glass [8, 9]. Defects such as micro-cracks and chippings are prone to be generated during laser processing as glass is brittle, seriously affecting the processing quality [9, 10]. Monitoring laser processing of float glass can help understand how the laser affects glass and predict whether defects are likely to occur. However, there is little research focusing on it so far.

Acoustic emission (AE) is a vibration phenomenon in a matrix material when phase transition or material removal happens under stress [11]. AE technique is a suitable and efficient method for monitoring the machining process, since it is a non-destructive testing method, and AE signals generated during the machining process include essential information about the machined materials [12, 13]. In the case of traditional machining methods, such as turning and milling, some researchers applied the AE technique to monitor the degree of metal material removal under different conditions [14–16]. Others studied the tool wear by extracting representative features from the AE signals [17–19]. In addition, there is a great deal of research about monitoring laser welding and additive manufacturing. The AE technique has been applied in some laser welding research to study the influence of different parameters such as laser power, welding speed, and depth of focus on the welding quality [20–22]. In other studies, AE has been used as a tool for predicting welding defects such as hanging slags [23–25]. When applied in additive manufacturing, the AE technique presents more advantages in terms of monitoring [26–28], such as indicating processing quality in laser powder bed fusion [29], identifying processing state, diagnosing defects in laser cladding [30], classifying defects in selective laser melting [31, 32], and so on. Almost all of the studies mentioned above have worked on AE monitoring of continuous laser processing of metal materials, and very little work has focused on pulsed laser processing [33, 34].

Since the AE monitoring technique has been successfully applied in various machining fields, it seems feasible to monitor pulsed laser processing. We conducted a nanosecond laser dotting experiment on float glass and applied the AE technique. Parameters were set differently to generate different laser dotting results: ablated pits and irregular cracks of various sizes. The AE analysis based on a method that combines framework and frame energy calculation effectively revealed multiple aspects of the laser ablation and crack generation. Thus, the main contribution of this work is the proposal of an intelligent AE monitoring method and the successful characterization of the process of laser dotting on float glass. In addition, the proposed method can be an alternative for AE monitoring of laser processing of other brittle materials.

The rest of this paper is organized as follows: Section 2 introduces the experiment system and design of experimental programs, Section 3 provides the AE analysis methods, experimental results and the corresponding AE analysis are shown in Section 4, and several vital conclusions are summarized in Section 5.

## Experiment

### Experimental system

**Fig. 1:**
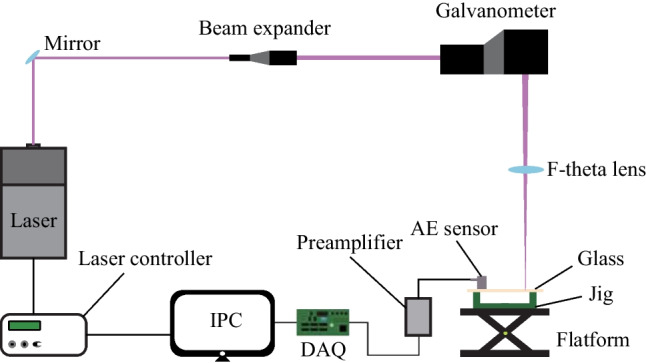
Schematic diagram of the experimental system

The laser dotting experimental system as schematically shows in Fig. 1 comprises a UV nanosecond laser (Poplar-355-5, China), a reflecting mirror, a beam expander, a scanning galvanometer (intelliSCAN 14, German), and an F-theta lens. The UV laser beam is generated from the laser source, reflected by the mirror, passes through the beam expander, and enter the galvanometer scanner. Then the laser beam is focused on the upper face of the float glass sample by the F-theta lens. The processing parameters of laser dotting could be set and adjusted in an industrial computer (IPC). The float glass sample is placed on a concave jig to ensure that the laser dotting was without disturbance.

The AE monitoring system includes an AE sensor (RS-45a, China), a preamplifier (PXA3, China), a data acquisition card (DAQ), and the IPC. The AE sensor is fixed on the upper face of the glass sample with an application of a coupling agent (PXUAC, China), and the frequency range of the AE sensor is 100–950 kHz. The preamplifier is used to effectively improve the signal-to-noise ratio. Data acquisition and signal processing are conducted by the IPC.

### Experimental design and characterization of laser dotting

The UV nanosecond laser used in this experiment is set in an external control mode, and the specific parameters which influence the dotting performances are identified as the pulse duration (*T*) and laser power (*P*) in a preliminary experiment. To further investigate the interaction between the UV nanosecond laser pulse and the float glass, we design a systematic experiment that apply AE monitoring. Both pulse duration and laser power are set at four different levels, and the specific parameters of laser dotting are listed in Table 1. After reviewing the relevant literature, the frequency range of AE signals for laser processing of float glass is found to be 150–350 kHz [35, 36]. To ensure that the amplitude of the sampled signal is not distorted, the sampling rate (*F*_s_) of the DAQ (PCI-1714U, ADVANTECH, China) is set as 5 MHz in the experiment.

**Table 1:** Specific values of laser dotting parameters

Experiment No.	*P*_1_ = 2.76 W	*P*_2_ = 5.08 W	*P*_3_ = 6.38 W	*P*_4_ = 7.45 W
*T*_1_ = 500 μs	*T*_1_*P*_1_	*T*_1_*P*_2_	*T*_1_*P*_3_	*T*_1_*P*_4_
*T*_2_ = 1000 μs	*T*_2_*P*_1_	*T*_2_*P*_2_	*T*_2_*P*_3_	*T*_2_*P*_4_
*T*_3_ = 1500 μs	*T*_3_*P*_1_	*T*_3_*P*_2_	*T*_3_*P*_3_	*T*_3_*P*_4_
*T*_4_ = 2000 μs	*T*_4_*P*_1_	*T*_4_*P*_2_	*T*_4_*P*_3_	*T*_4_*P*_4_

**Fig. 2:**
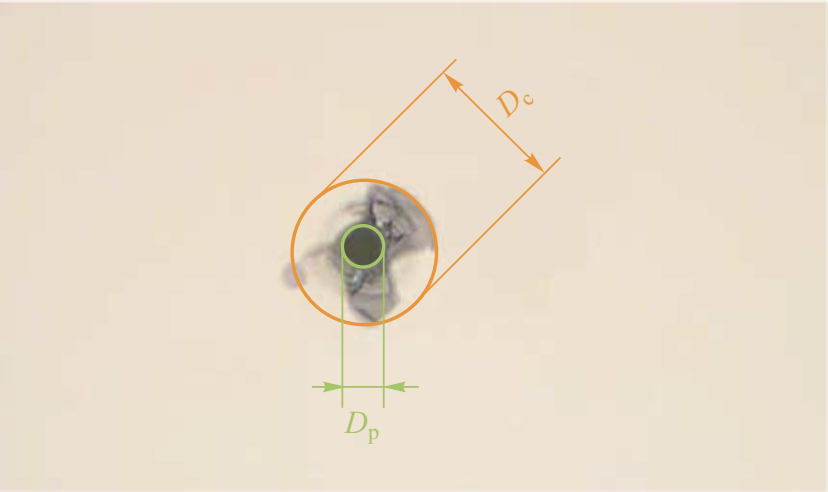
Typical image of laser dotting and its measurement

After laser dotting, the float glass samples are ultrasonically cleaned in ethanol and deionized water in turn, and then dried at room temperature. We apply an electronic microscope to observe the surface topography after laser dotting, and the typical image is shown in Fig. 2. A nearly circular ablation pit can be observed in the figure, together with some irregular cracks around the ablation pit. We measure the diameter of the ablation pit (${D}_{\text{p}}$) to characterize the ablation intensity, and the maximum diameter of the cracks (${D}_{\text{c}}$) to reveal the degree of instability of the laser dotting. The final ${D}_{\text{p}}$ and ${D}_{\text{c}}$ values are averaged over 10 measurements, to eliminate observation errors.

## AE analysis

A raw AE signal is a one-dimensional data with equal time intervals, and the data length is usually large since the sampling frequency of the DAQ is relatively high to monitor the process better. Windowed segmentation and characteristic parameter analysis based on segmented signals are the most common methods to reveal the processing state on the time scale. The ordinary windowed segmentation with constant window length may sometimes ignore significant data point at the end of the window. In this section, we introduce a method that combines framework with energy calculation to extract features in the AE signals of laser dotting processing.

### Framework in AE signals

**Fig. 3:**
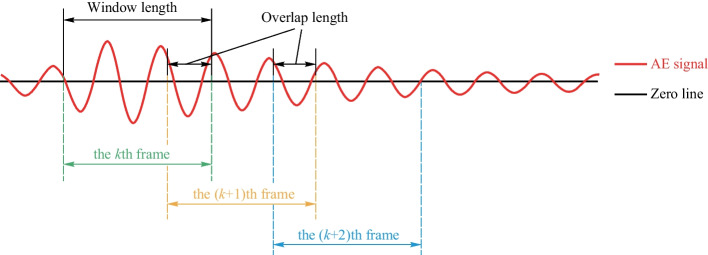
Schematic diagram of the framework in AE signal

Framework is a mature tool for dealing with the natural language processing problem, as it overcomes the instability of speech signals [37]. The main idea of the framework is to apply a sliding window to segment the raw signal and an appropriate window function to eliminate the leakage at the endpoint of the window. As illustrated in Fig. 3, the raw AE signal is drawn in red, and it is zero-crossing vibrated. When applying the framework in AE analysis, the window length (${w}_{l}$) and the overlap length (${o}_{l}$) should be set first. Figure 3 shows the selection of the $k$, $(k+1)$, and $(k+2){\text{th}}$ frames. Here the formula for these frames is presented as follows:

1 $$\begin{aligned}f(k) & =\left[{x}_{j},{x}_{j+1},\ldots ,{x}_{j+wl-1}\right], \\ f\left(k+1\right) & =\left[{x}_{j+wl-ol},\ldots ,{x}_{j+2wl-ol-1}\right],\\ f(k+2) & =\left[{x}_{j+2wl-2ol},\ldots ,{x}_{j+3wl-2ol-1}\right],\end{aligned}$$

where $f(k)$, $f(k+1)$, and $f(k+2)$, are the frames extracted from the raw signal sequence of ${\varvec{X}}=[{x}_{1},{x}_{2},{x}_{3},\ldots ,{x}_{L}]$. Assuming that the term ${x}_{j}$ is the first element in frame $f(k)$, then the other element of frames $f(k)$, $f(k+1)$, $f(k+2)$ … can be obtained Eq. (1). The raw AE signal is transformed into a frame sequence with equal length, and all data points in the raw AE signal are continuous except for the starting/ending point. Analyzing the AE signal with an application of the framework seems appropriate and accurate.

### Energy calculation in frame

After the application of the framework, the object of signal analysis is transformed from the raw signal to the frame sequence. The character energy can eliminate the zero-crossing vibration and reveal the intensity of the raw AE signal [38]. Due to the advantage of this method, we calculate the energy of each frame to analyze the raw AE signal. Assuming $f(k)=[{x}_{m},{x}_{m+1},{x}_{m+2},\ldots ,{x}_{n}]$ is the ${k}{\text{th}}$ frame of a raw AE signal with data length of *L* recorded as of ${\varvec{X}}=[{x}_{1},{x}_{2},{x}_{3},\ldots ,{x}_{L}]$. The energy of $f(k)$ is defined as

2 $${E}_{k}=\sum_{m}^{n}{x}_{i}^{2}, \space i=m,m+1,m+2,\ldots ,n.$$

This formula transforms the zero-crossing vibrated raw AE signal into a non-negative energy sequence. Since laser dotting is a continuous-time process, the calculated energy sequence changes with the time. It is necessary to extract characteristic parameters to reveal the changing trend of the calculated energy sequence.

**Fig. 4:**
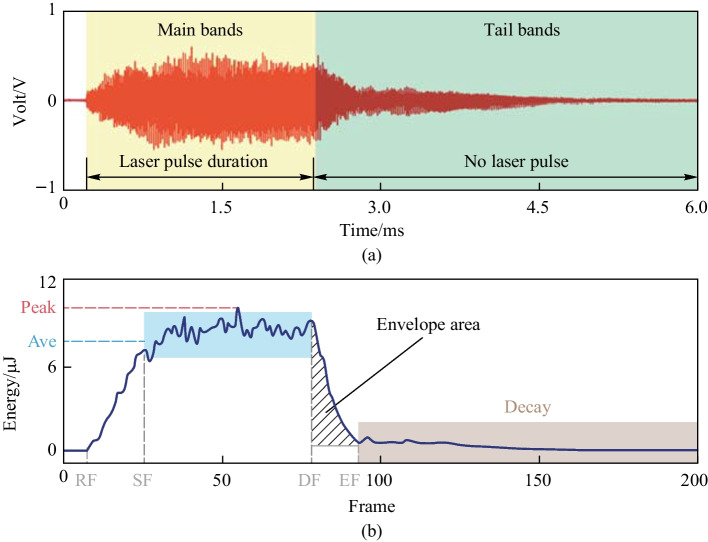
Typical AE signal of **a** laser dotting and **b** its corresponding framework energy

As shown in Fig. 4a, the typical AE signal of laser dotting was zero-crossing vibrated, and we manually split the raw AE signal into two bands (the main and tail bands) based on the laser pulse duration. The intensity of the AE signal in the main bands was significantly larger than that in the tail bands. By applying framework and energy calculation, the energy curve as shown in Fig. 4b was obtained; we extracted some characteristic parameters to describe the curve feature. Compared with the raw AE signals in Fig. 4a, the energy curve shows a similar changing trend as the amplitude of the raw AE signal; it increased sharply at the rising frame (RF), stated to be stabilized gradually at the stable frame (SF), started to decrease at the falling frame (FF), and nearly reduced to zero at end frame (EF). Except for the four special frames, we extracted the maximum energy value (Peak) and the average energy value (${\text{AveEng}}$) between the SF and FF as the characteristics in the main bands. The envelope area of the energy curve between FF and EF was calculated to describe the signal intensity in the tail band.

## Results and discussion

### Laser dotting results

**Fig. 5:**
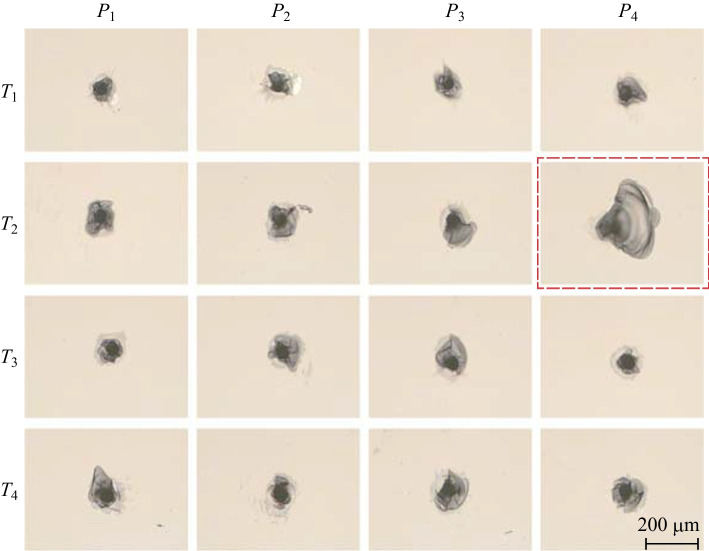
UV nanosecond laser dotting results for different parameters in the experiment as given in Table 1

The laser dotting experiment with different laser power and duration was conducted, and the microscope images of laser dotting results are shown in Fig. 5. The topography of all dotting results present ablated pits with irregularly shaped cracks distributed around pits, and the corresponding measurements of ${D}_{\text{p}}$ and ${D}_{\text{c}}$ are conducted, and the outcomes are listed in Table 2. The values of ${D}_{\text{p}}$ and ${D}_{\text{c}}$ are different as the laser power and duration vary. In addition, the dotting result of experiment No. ${T}_{2}{P}_{4}$ is marked within a red box in Fig. 5 because the crack was abnormally larger than the others.

**Table 2:** Measurement results of the laser dotting experiment

Experiment No.	${D}_{\text{p}}$		${D}_{\text{c}}$	
	Ave/μm	Std	Ave/μm	Std
${T}_{1}{P}_{1}$	48.505	1.343	102.759	30.547
${T}_{1}{P}_{2}$	52.466	1.443	94.233	17.084
${T}_{1}{P}_{3}$	54.825	1.562	105.274	29.247
${T}_{1}{P}_{4}$	58.005	2.208	96.622	25.322
${T}_{2}{P}_{1}$	53.612	1.810	89.354	13.446
${T}_{2}{P}_{2}$	57.315	2.451	107.049	11.448
${T}_{2}{P}_{3}$	59.298	2.682	101.040	31.279
${T}_{2}{P}_{4}$	61.721	3.139	139.841	61.248
${T}_{3}{P}_{1}$	54.824	1.839	82.740	9.196
${T}_{3}{P}_{2}$	59.298	2.790	110.032	16.873
${T}_{3}{P}_{3}$	60.504	2.792	88.245	27.068
${T}_{3}{P}_{4}$	63.129	3.970	82.892	29.855
${T}_{4}{P}_{1}$	58.005	1.993	100.368	26.027
${T}_{4}{P}_{2}$	61.309	2.806	86.176	13.714
${T}_{4}{P}_{3}$	63.309	3.337	88.794	13.351
${T}_{4}{P}_{4}$	67.226	4.211	95.490	10.980

**Fig. 6:**
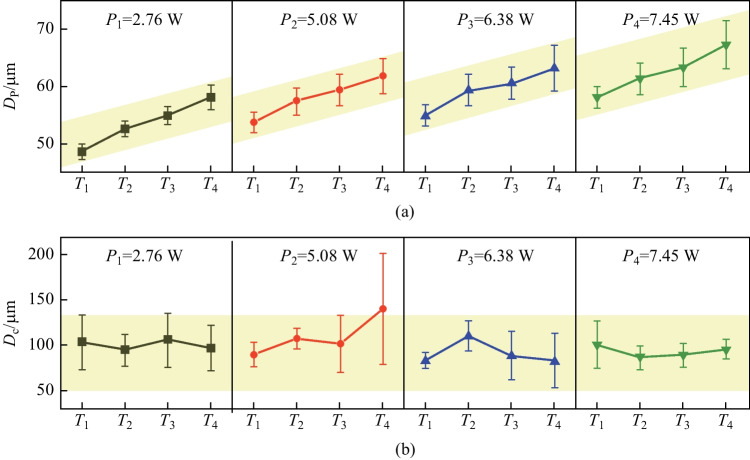
Measurement results of **a** ${D}_{\text{p}}$ and **b** ${D}_{\text{c}}$

The measurement results (average values and standard deviations of ${D}_{\text{p}}$, ${D}_{\text{c}}$) of laser dotting under different laser power and duration are shown in Fig. 6. The most significant value of ${D}_{\text{p}}$ (67.226 μm) obtained in experiment No. ${T}_{4}{P}_{4}$, and the smallest value (48.505 μm) obtained in experiment No. ${T}_{1}{P}_{1}$, as shown in Fig. 6a. Overall, the value of ${D}_{\text{p}}$ rose gradually with increasing laser power and duration. Specifically, the ${D}_{\text{p}}$ values under different laser powers showed a similar upward trend when the duration increased from *T*_1_ (500 μs) to *T*_4_ (2000 μs). In addition, the standard deviation of *D*_p_ also showed a positive correlation with the laser power and duration. The ${D}_{\text{c}}$ value indicated a relatively stable state of distribution, as illustrated in Fig. 6b. The ${D}_{\text{c}}$ values for different combinations of laser power and duration mainly distributed between 80 and 110 μm. However, a substantial value (139.841 μm) obtained in experiment No. of ${T}_{2}{P}_{4}$. In addition, the standard deviations of ${D}_{\text{c}}$ mostly distributed in the range of 10–30, while the standard deviations of ${D}_{\text{c}}$ of experiment No. ${T}_{2}{P}_{4}$ had the largest value of 61.248.

The measurement results of ${D}_{\text{p}}$ reveal that the degree of laser ablation become stronger with increasing laser power and duration. On the one hand, the mean value of ${D}_{\text{p}}$ gradually rose, demonstrating that the average intensity of laser ablation increases. On the other hand, the standard deviation also increased, indicating that the ablation intensity became more unstable when laser power and duration were at larger values. The measurement results of ${D}_{\text{c}}$ show that there seemed to be little relationship between the geometric size of the cracks and the processing parameters. Despite the enormous crack in experiment No. ${T}_{2}{P}_{4}$, average values and standard deviation of *D*_c_ changed little with varying parameters.

### Correlation analysis between laser ablation and AE signals in the main band

The AE signals acquired in the laser dotting experiment are divided into the main and tail bands according to the pulse duration. The AE signals in the main band can directly reveal the laser ablation process because the interaction between the laser pulse and float glass happens at the same time. Since the laser dotting experiments are designed with different pulse duration and power, whether the AE analysis can reveal the time and intensity of laser ablation is the first problem to be concerned. The frame duration ($\text{FD}=\text{FF}-\text{SF}$) and the average energy (Ave) of AE signals in the main band are calculated to explore the above problem. In the signal acquisition step, the length of each signal is set to ${L}_{\text{S}}=30000$. In the AE’s framework analysis, the key parameters ${w}_{l}$ and ${o}_{l}$ are 200 and 50, respectively.

**Fig. 7:**
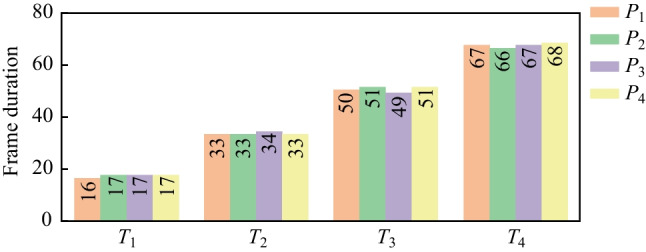
Time duration calculation in the main band of AE signals

As shown in Fig. 7, the length of the main band in the AE signal is distributed in a relatively concentrated manner for different pulse durations, and the frame duration gradually rises with the increase of pulse duration. The frame duration is more like an ascending stair-like distribution and correlates with the pulse duration. The frame duration can be transformed into the time scale ($T_\text{d}=\text{FD}*({w}_{l}-{\text{o}}_{l})/F_{\text{s}}$), as presented in Table 3. The calculated time durations nearly equal the corresponding theoretical value in the dotting experiment. Undeniably, some errors exist in the calculated time duration compared to the actual pulse duration, which may be generated by the framework and errors in the extraction of characteristic parameters from the energy curves.

**Table 3:** Calculated interaction time based on frame durations

Experiment No.	Calculated interaction time duration/μs	Pulse duration in the experiment/μs
${T}_{1}{P}_{1}$	480	500
${T}_{1}{P}_{2}$	510	500
${T}_{1}{P}_{3}$	510	500
${T}_{1}{P}_{4}$	510	500
${T}_{2}{P}_{1}$	990	1000
${T}_{2}{P}_{2}$	990	1000
${T}_{2}{P}_{3}$	1020	1000
${T}_{2}{P}_{4}$	990	1000
${T}_{3}{P}_{1}$	1500	1500
${T}_{3}{P}_{2}$	1530	1500
${T}_{3}{P}_{3}$	1470	1500
${T}_{3}{P}_{4}$	1530	1500
${T}_{4}{P}_{1}$	2010	2000
${T}_{4}{P}_{2}$	1980	2000
${T}_{4}{P}_{3}$	2010	2000
${T}_{4}{P}_{4}$	2040	2000

**Fig. 8:**
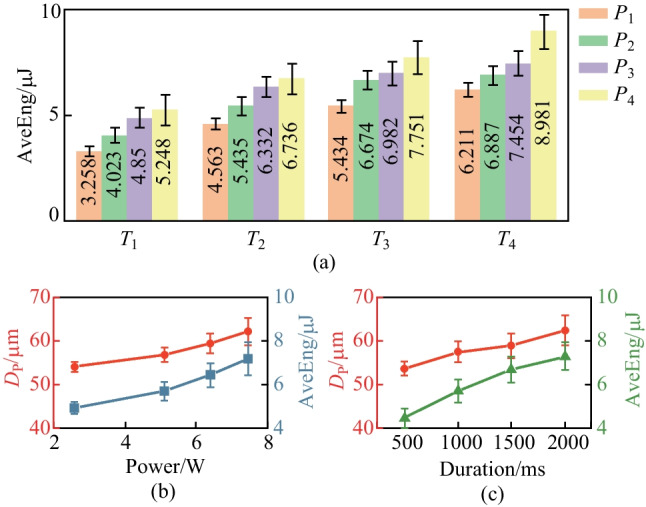
**a** Average energy for different parameters; **b** and **c** show the correlation between framework average energy and ${D}_{\text{p}}$

We calculate the average value of energy ($\text{AveEng}$) between the SF and FF in a frame of AE signal to study the correlation between AE signals and the intensity of laser ablation. The average values of the energy are illustrated in Fig. 8a. The $\text{AveEng}$ rises with increasing laser power under different pulse durations. Moreover, it also presents a similar ascending trend when only considering the influence of the pulse duration. The minimum value of $\text{AveEng}$ occurs at the experiment No. ${T}_{1}{P}_{1}$, while the maximum value happens in the case of ${T}_{4}{P}_{4}$. The distribution and changing trends of $\text{AveEng}$ values are similar to the ${D}_{\text{p}}$ measurement results, and we conduct further analysis to study the correlation between $\text{AveEng}$ values and the ${D}_{\text{p}}$ measurements. Figure 8b shows that the ${D}_{\text{p}}$ and $\text{AveEng}$ rise with similar trends when the laser power increases. In the meantime, the standard deviations of ${D}_{\text{p}}$ and $\text{AveEng}$ also increase when the laser power rises. When laser pulse power is unchanged, the* D*_p_ and $\text{AveEng}$ ascend with larger standard deviations when the pulse duration increases, as shown in Fig. 8c. A significant correlation exists between the $\text{AveEng}$ values and the ${D}_{\text{p}}$ measurements regardless of whether laser power and pulsed duration are considered.

Monitoring AE signal can effectively reveal the laser ablation process in the laser dotting experiment by extracting the characteristic parameters from the energy curve in the main band. Respectively, the frame duration reveals the interaction time of laser dotting, and the average values of energy show a significant correlation with the geometric size of ablated pits. Therefore, the AE analysis in the main bands can fully interpret the laser ablation effect in laser dotting in terms of both duration and intensity.

### Cracks analysis using AE signals in tail band

The AE signals in the tail band can be regarded as the natural decay of main band signals once no other events occur. During the laser dotting process, the laser ablation only happens in the laser pulse duration represented by the AE signals in the main band, and thermal diffusion behavior is the prominent source of signals in the tail band. Analysis of the characteristic parameters of frame duration and average energy values shows that the crack phenomenon does not directly relate to the main band. We calculate the envelope area of the energy curve in the tail band to discover the relation between cracks and AE signals in the tail band.

**Fig. 9:**
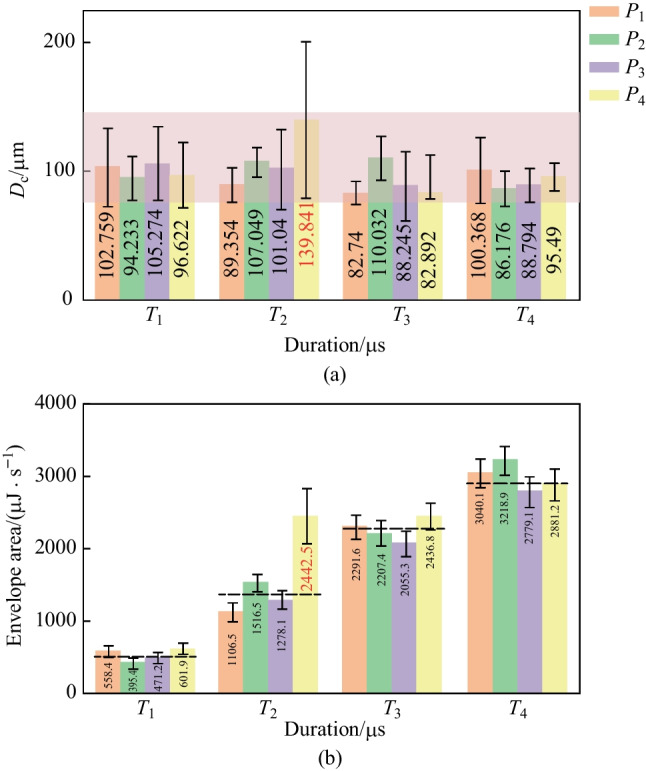
**a** *D*_c_ measurements and **b** envelope area calculation of AE signals in the tail bands

We illustrate histograms of *D*_c_ and envelope area to better reveal the characteristics in the tail band. The maximum diameters of irregular cracks are concentrated in a range of 80–110 μm, as illustrated in Fig. 9a. The histogram of the envelope area is shown in Fig. 9b, and it presents a gradual ascending stair-like trend with increasing pulse duration. The standard deviations of *D*_c_ and envelope area are relatively stable when the processing parameters change. Specifically, the standard deviations of ${D}_{\text{c}}$ are distributed in a range of 10–30, and the standard deviation of envelope area is 70–200. The enormous crack size (139.841 μm) with a huge standard deviation (61.248 μm) occurs with the experiment No. of ${T}_{2}{P}_{4}$. In the meantime, the envelope area also has a significantly large value (2442.5 μJ s) with a huge standard deviation (380.878).

It is easy to understand that the laser ablation is more intense when the pulse duration increases. In the meantime, the energy of AE signals in the main band increases substantially, which results in a larger envelope area value in the tail band. The distribution of envelop area is relatively concentrated for pulse durations of *T*_1_,* T*_3_, and *T*_4_. While an exception happens for the pulse duration of *T*_2_, the envelope area in the case of of ${T}_{2}{P}_{4}$ is much larger than the others, corresponding to the crack with an enormous size. Therefore, the measurement results of ${D}_{\text{c}}$ and the calculated results of envelope area present a high consistency.

**Fig. 10:**
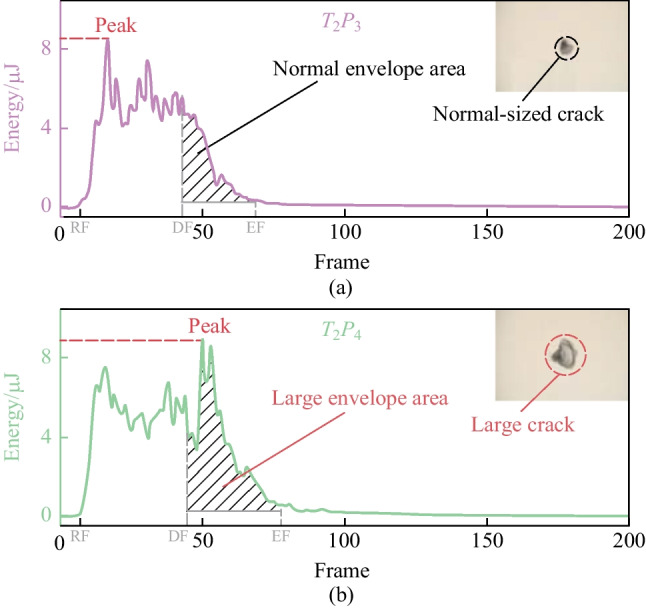
Specific energy curves under parameter combinations of **a** ${T}_{2}{P}_{3}$, and **b** ${T}_{2}{P}_{4}$

We select the specific laser dotting parameter combinations of ${T}_{2}{P}_{3}$ and ${T}_{2}{P}_{4}$ for the comparative analysis of the AE signal characteristics concerning the very large cracks. The corresponding energy curves are shown in Fig. 10. Although the laser processing parameters are close, the energy curves are different. As presented in Fig. 10a, the energy curve of experiment ${T}_{2}{P}_{3}$ shows a normal trend, with rise at the RF, decline at FF, and decay at EF. The energy curve of experiment ${T}_{2}{P}_{4}$ is significantly different. The curve shows a sharp increase after the FF, where laser ablation has already ended, as shown in Fig. 10b. In addition, the peak values of the two energy curves are almost the same, but the locations of peak values are different. The peak value of experiment ${T}_{2}{P}_{3}$ is in the main band, while the other is in the tail bands, causing a large envelope area. Since no laser ablation happens in the tail bands, significantly large cracks existing in the experiment ${T}_{2}{P}_{4}$ indicate that the growth of large cracks may be related to a large envelope area.

To summarize, the envelope area of the energy curve in the tail band is calculated to extract the feature of significantly large cracks. Both the width of cracks and the envelope area of energy curves are generally stable and show a noticeable change in the experiment ${T}_{2}{P}_{4}$, indicating that the envelope area can reflect the crack phenomenon in laser dotting. Different characteristics of energy curves are exhibited by comparing experiments ${T}_{2}{P}_{3}$ and ${T}_{2}{P}_{4}$. Experiment ${T}_{2}{P}_{3}$ shows a classic and stable energy curve; while ${T}_{2}{P}_{4}$ is aberrant. The characteristics of the energy curve for experiment ${T}_{2}{P}_{4}$, such as the sharp increase after FF and the peak location in the tail bands, indicate that the generation of cracks happens after the pulse duration.

## Conclusions

This paper introduced an analysis method to reveal the pulsed laser interaction mechanism based on the AE monitoring, which was demonstrated by designing an experiment of a nanosecond ultraviolet laser dotting on float glass. A method that combined framework and frame energy calculation was used in the AE analysis to explore the laser dotting process from the AE signal. Characteristics of the energy curves in the main band can be used to effectively evaluated laser ablation in the pulse duration, and the crack generation can be determined by analyzing features in the tail band. The whole process of laser dotting on float glass was successfully monitored, and the interaction between the pulsed laser and float glass was elaborated clearly. Several vital conclusions were summarized as follows:

1. Characteristic parameters (frame duration and average value of energy) in the main band could be used to successfully evaluate the laser ablation. The frame duration presented a stair-like rising trend with increase in pulse duration, and the average value of energy showed a significant correlation to the geometric size of ablation pits. The analysis of characteristic parameters in the main band can reveal the properties of laser ablation in terms of interaction time and intensity.
2. The envelope area of the energy curve in the tail band was calculated to reveal crack generation. The envelope area was stable in most cases but presented a noticeable change when the large cracks occurred. The characteristic of the energy curve in the tail band also demonstrated that crack generation happened after laser ablation.
3. The proposed AE monitoring method could successfully reveal laser ablation and crack generation in the process of nanosecond laser dotting on float glass. The method can be also used to explore the interaction mechanism in laser processing of other brittle materials.

## Data Availability

The data that support the findings of this study are available from the corresponding author, upon reasonable request.
